# Anti-pancreatic tumor efficacy of a Listeria-based, Annexin A2-targeting immunotherapy in combination with anti-PD-1 antibodies

**DOI:** 10.1186/s40425-019-0601-5

**Published:** 2019-05-22

**Authors:** Victoria M. Kim, Alex B. Blair, Peter Lauer, Kelly Foley, Xu Che, Kevin Soares, Tao Xia, Stephen T. Muth, Jennifer Kleponis, Todd D. Armstrong, Christopher L. Wolfgang, Elizabeth M. Jaffee, Dirk Brockstedt, Lei Zheng

**Affiliations:** 1 The Sidney Kimmel Comprehensive Cancer Center, The Johns Hopkins University School of Medicine, Baltimore, MD 21287 USA; 2 Department of Oncology, The Johns Hopkins University School of Medicine, Baltimore, MD 21287 USA; 3 Department of Surgery, The Johns Hopkins University School of Medicine, Baltimore, MD 21287 USA; 4 The Pancreatic Cancer Precision Medicine Program of Excellence, The Johns Hopkins University School of Medicine, Baltimore, MD 21287 USA; 5 Aduro Biotech, Inc., Berkeley, California USA

**Keywords:** Pancreatic cancer, Immunotherapy, Annexin A2, Anti-PD-1 antibody, interferon-gamma, CD8 T cells

## Abstract

**Background:**

Immune checkpoint inhibitors are not effective for pancreatic ductal adenocarcinoma (PDAC) as single agents. Vaccine therapy may sensitize PDACs to checkpoint inhibitor treatments. Annexin A2 (ANXA2) is a pro-metastasis protein, previously identified as a relevant PDAC antigen that is expressed by a GM-CSF-secreting allogenic whole pancreatic tumor cell vaccine (GVAX) to induce an anti-ANXA2 antibody response in patients with PDAC. We hypothesized that an ANXA2-targeting vaccine approach not only provokes an immune response but also mounts anti-tumor effects.

**Methods:**

We developed a Listeria-based, ANXA2-targeting cancer immunotherapy (Lm-ANXA2) and investigated its effectiveness within two murine models of PDAC.

**Results:**

We show that Lm-ANXA2 prolonged the survival in a transplant model of mouse PDACs. More importantly, priming with the Lm-ANXA2 treatment prior to administration of anti-PD-1 antibodies increased cure rates in the implanted PDAC model and resulted in objective tumor responses and prolonged survival in the genetically engineered spontaneous PDAC model. In tumors treated with Lm-ANXA2 followed by anti-PD-1 antibody, the T cells specific to ANXA2 had significantly increased INF**γ** expression.

**Conclusions:**

For the first time, a listeria vaccine-based immunotherapy was shown to be able to induce a tumor antigen-specific T cell response within the tumor microenvironment of a “cold” tumor such as PDAC and sensitize the tumor to checkpoint inhibitor therapy. Moreover, this combination immunotherapy led to objective tumor responses and survival benefit in the mice with spontaneously developed PDAC tumors. Therefore, our study supports developing Lm-ANXA2 as a therapeutic agent in combination with anti-PD-1 antibody for PDAC treatment.

**Electronic supplementary material:**

The online version of this article (10.1186/s40425-019-0601-5) contains supplementary material, which is available to authorized users.

## Background

Pancreatic ductal adenocarcinoma (PDAC) is a highly mortal disease. While it accounts for only 3% of estimated new cancer cases each year, it is currently the fourth most common cause of cancer mortality in the United States [1]. By 2030, it is expected to be the 2nd leading cause of cancer death in the U.S. surpassing breast and colorectal cancer [2]. The overall 5-year survival rate is only 20% for patients with localized disease amenable to surgical resection and a mere 2% for those with distant metastasis [3]. This attests to the aggressive biology of PDAC as well as the demand for a new modality of therapy.

Immunotherapy has been a monumental advancement over the last decade for the treatment of cancer. Programmed death-1 (PD-1) and its ligand, PD-L1, are cell surface markers involved in regulatory checkpoint pathways and have been targets for blockade in the immunotherapy of many solid tumor types [4, 5]; however, these anti-PD-1 or PD-L1 antibodies have limited activity for PDAC as single agents [6]. Nevertheless, our preclinical and clinical research has suggested that a vaccine therapy, particularly the GM-CSF-secreting pancreatic tumor whole cell vaccine (GVAX), can re-program the tumor microenvironment and prime PDAC for anti-PD-1 or PD-L1 therapies [7–9]. Thus, PDAC can be sensitized to checkpoint inhibitor blockade, providing a rationale to use these agents in combination despite their limited anti-tumor effect as single agent therapy. Unfortunately, a whole cell vaccine is limited by only a small portion of antigens being immunogenic and thus the expression of immunogenic antigens being insufficient. Antigen-specific vaccines, such as alpha-enolase targeted DNA vaccination, have been shown to induce coordinated anti-tumor, T cell based immune responses in preclinical models of PDAC [10]. However, the priming effect of antigen-specific vaccines for checkpoint inhibitors has not been well described.

We previously identified mesothelin as an immune dominant antigen presented by GVAX [11–13]. CRS-207, a mesothelin-expressing Listeria-based immunotherapy, has been developed by using a Listeria-based vector [14]. More recently, mesothelin was also shown to be an antigen for T cell therapy that may elicit an effective intratumoral immune response in the mouse model of PDAC [15]. Although CRS-207, when given sequentially with GVAX, improved survival comparing to GVAX alone [16], it failed to show its benefit over chemotherapy. Thus, there is a need to investigate additional immune dominant antigens of PDAC. We identified ANXA2 as a pro-metastasis, PDAC-associated protein through sero-proteomic analysis of sera from patients who were responding to GVAX [17, 18]. In contrast to mesothelin, for which limited data supports its biological role in PDAC development, ANXA2 is biologically essential for PDAC metastasis and the development of many other cancer types [17, 19–23]. Previously, an immune independent, direct targeted effect of anti-ANXA2 antibody was seen on suppressing metastases [17]. Because ANXA2 is a functionally relevant protein, specifically in tumor cells with a high metastatic potential, it is conceivable that PDAC tumor cells would not be able to evade antitumor T cell immunity through antigen loss if ANXA2 is the antigen used for a Listeria-based immunotherapy.

In this study, we showed that the ANXA2-expressing Listeria-based immunotherapy (Lm-ANXA2) prolonged survival in the mouse model of PDACs and the sequential treatment with Lm-ANXA2 followed by anti-PD-1 therapy increased the interferon γ (INFγ) expression of an ANXA2 epitope specific T cell response within the tumor microenvironment.

## Study methods

### Cell lines and medium

KPC tumor cells are a syngeneic PDAC line derived from genetically engineered mice having tissue-specific KRAS and p53 knock-in mutations [24]. They were developed and cultured as described previously [19, 25]. KPC cells used were at passage 4 to 5 and cultured for 2 to 3 additional passages if necessary. KPC cells were maintained in RPMI (LifeTechnologies), 10% fetal bovine serum (Atlas Biologicals), 1% L-glutamine (Life Technologies), 1% penicillin/streptomycin (LifeTechnologies), 1% sodium pyruvate (LifeTechnologies), 1% nonessential amino acids (LifeTechnologies) at 37 °C in 5% CO_2_.

Murine H-2 Kb and H-2 Db T2 cells were maintained in RPMI (LifeTechnologies), 10% fetal bovine serum (Atlas Biologicals), 1% L-glutamine (LifeTechnologies), 0.5% penicillin/streptomycin (LifeTechnologies), 1% sodium pyruvate (LifeTechnologies), 1% nonessential amino acids (LifeTechnologies), and 0.2% G418 (Thermofisher) at 37 °C in 5% CO_2_. The cell lines were validated by STR profiling and tested for mycoplasma every 6 months.

### Human ANXA2 peptides

A panel of 339 peptides was synthesized as a means to identify Kb- and Db-restricted murine epitopes. Murine and human ANXA2 are 98% homologous (Additional file 1: Figure S1). The Peptide Generator Database (PeptGen) (https://www.hiv.lanl.gov/content/sequence/PEPTGEN/peptgen.html) was used to create sets of overlapping peptides of the human ANXA2 protein sequence obtained from the NCBI Protein database. Consecutive peptides were constructed as 15-mers that overlapped by 10 (> 95% purity) at the Johns Hopkins Oncology Peptide Synthesis Core Facility. 15-mer peptides were pooled into 4 initial groups (Additional file 1: Table S1). Following identification of a pooled peptide group of interest, individual 15-mer peptides were tested for activity. Consecutive 8-mer peptides were then constructed from identified active 15-mer sequences, overlapped by 7 amino acids (> 90% purity) (Peptide 2.0 Inc.)

### Construction of lm-ANXA2

The Lm-ANXA2 strain was constructed in the Δ*actA* Δ*inlB* live attenuated, double deleted (LADD) *Listeria* platform strain [26]. The ANXA2 coding sequence was synthesized using *Listeria* codon bias (ATUM, Newark, CA), and cloned downstream the *actA* promoter and in-frame with amino terminus of ActA in a derivative of the pPL2 integration vector [27]. The plasmid construct was stably integrated at the *tRNA*^Arg^ locus as described. An empty LADD strain was used as a control (Empty Lm).

### Intracellular Western blot

Expression and secretion of ANXA2 were performed in DC2.4 cells essentially as described [28]. ActA fusions (ANXA2) were probed with a polyclonal antibody against the mature amino terminus of the secreted ActA protein. Expression was stable after infection and was normalized to the Listerial P60 protein using the monoclonal antibody P6007 (EMDMillipore). IRDye secondary antibodies were used per manufactures instructions and images were captured using an Odyssey Imaging System (Licor Biosciences).

### Mice and in vivo experiments

Six- to eight-week old C57Bl6 female mice were purchased from Harlan Laboratories (Frederick, MD) and maintained in accordance with the Institutional Animal Care and Use Committee (IACUC) guidelines. According to the IACUC mouse protocol, mice with signs of distress including hunched posture, lethargy, dehydration, and rough hair coat would be euthanized and considered to have reached the “survival” endpoint. The hemispleen technique of tumor inoculation was performed on Day 0 as previously described [30]. Briefly, after anesthetization of the mouse, the spleen is eviscerated, clipped, and divided in half. One half of the spleen is injected with 2 × 10^5^ KPC tumor cells and the injected hemispleen is subsequently removed. This easily reproducible preclinical model allows consistent development of aggressive liver metastases, and is ideal for the immunologic investigation of metastatic disease within the liver microenvironment. On day 2, a single dose of cyclophosphamide (Cy) (100 mg/kg, Bristol-Myers Squibb) was administered intraperitoneally (IP). On days 3, 10, and 17, Empty Lm and Lm-ANXA2 (5 × 10^5^ colony-forming units) in 0.2 mL phosphate-buffered saline (PBS) was administered IP. Listeria dosing was initiated on day 3 after establishment of metastatic tumor burden as previously described [31]. Starting on day 24 Anti-PD-1 antibody (5 mg/kg IP, RMP1–14, BioXcell) or isotype control IgG (5 mg/kg IP, 2A3, BioXcell) were administered IP twice weekly until death or Day 90. For CD8+ T cell depletion, anti-CD8a antibody (10 mg/kg IP, 2.43, BioXcell) was started on day 2 and administered IP twice weekly until day 30.

Genetically engineered KPC mice (KRAS and p53 mutations) [24] were examined by a small animal ultrasound (Vevo770) weekly. Once PDAC tumors reached 3-5 mm in size, mice were randomized (day 0) to either of two treatment groups: Empty Lm vaccination followed by IgG antibody or Lm-ANXA2 followed by anti-PD-1 blockade. Listeria-based immunotherapy were administered on days 1, 8, and 15. Anti-PD-1 antibody (5 mg/kg) or IgG (5 mg/kg) were administered IP twice weekly for a total of 5 doses starting day 22.

### Analysis of the safety of Listeria

Six- to eight-week old C57Bl6 female mice were purchased and maintained as described above. On days 1, 8, and 15, Empty Lm and Lm-ANXA2 (5 × 10^5^ colony-forming units) in 0.2 mL PBS was administered IP. Weight in grams was measured and followed in each mouse. On day 18, serum was collected from each mouse and aspartate aminotransferase (AST) and alanine aminotransferase (ALT) levels were measured at SRI Biosciences’ Clinical Analysis Laboratory.

### Analysis of liver metastasis infiltrating lymphocytes

Analysis of liver metastasis-infiltrating lymphocytes was performed on day 28 after KPC tumor inoculation. Each liver was mechanically processed through 100-μm and 40-μm nylon filter and brought to a volume of 25 mL CTL medium. Each spleen was mechanically processed through 100-μm nylon filter and brought to a volume of 15 mL CTL medium. All suspensions were centrifuged at 1500 rpm for 5 min. Liver cell pellets were suspended in 4 mL of ACK lysis (Quality Biological,) and spleen cell pellets were suspended in 2 mL ACK lysis for 2 min and all were subsequently spun at 1500 rpm for 5 min. Liver cell pellets were then suspended in 5 mL 80% Percoll (GE Healthcare Life Sciences), overlaid with 5 mL 40% Percoll and centrifuged at room temperature for 25 min at 3200 rpm without brake. The lymphocyte layer was removed and suspended in 10 mL CTL media.

### Mouse IFNγ enzyme-linked immunosorbent and ImmunoSpot assays

Isolated liver infiltrating lymphocytes were enriched for CD8+ cells using CD8-negative isolation kits (LifeTechnologies) according to the protocol provided by the manufacturer. Peptide-stimulated Kb and Db cells were added to isolated CD8+ T cells at a ratio of 1:1 (2 × 10^5^ cells of each respective cell line) and incubated for 18 h at 37 °C. Mouse IFNγ Enzyme-Linked Immunosorbent Assay (ELISA) Ready-Set-Go assay was conducted per the manufacturer’s protocol (eBioscience). Mouse IFNγ Enzyme-Linked ImmunoSpot (ELISPOT) assay was conducted per the manufacturer’s protocol (Abcam).

### Quantitative real time reverse transcription polymerase chain reaction (RT-PCR)

TRIzol Reagent (ThermoFisher Scientific) was used to extract total RNA from tumor infiltrating lymphocyte cell pellets. The RNA was then converted to cDNA using the Superscript III First Strand Synthesis Supermix Kit (ThermoFisher Scientific). Quantitative real-time RT-PCR (qPCR) was performed on the StepOnePlus Real Time PCR System (ThermoFisher Scientific) and analyzed by the StepOne software V2.1. The expression of TNFα, IL-2, IFNα, IFNβ, and IFNγ was measured by SYBR Green-based qPCR. All gene expression was normalized to the expression of β-actin. All qPCR reactions were performed in triplicate.

### Statistical analysis

Statistical analyses for survival were conducted using Kaplan-Meier curves and log-rank tests for survival. Comparisons of cure rates were conducted using Fisher’s exact test. Comparison of mean cytokine expression between two groups was conducted using unpaired student’s t-test. Comparison of maximum tumor response was conducted using one-way ANOVA test for multiple comparisons. A *p* value of < 0.05 was considered statistically significant.

## Results

### Construction of ANXA2-expressing LADD strain

**Fig. 1:**
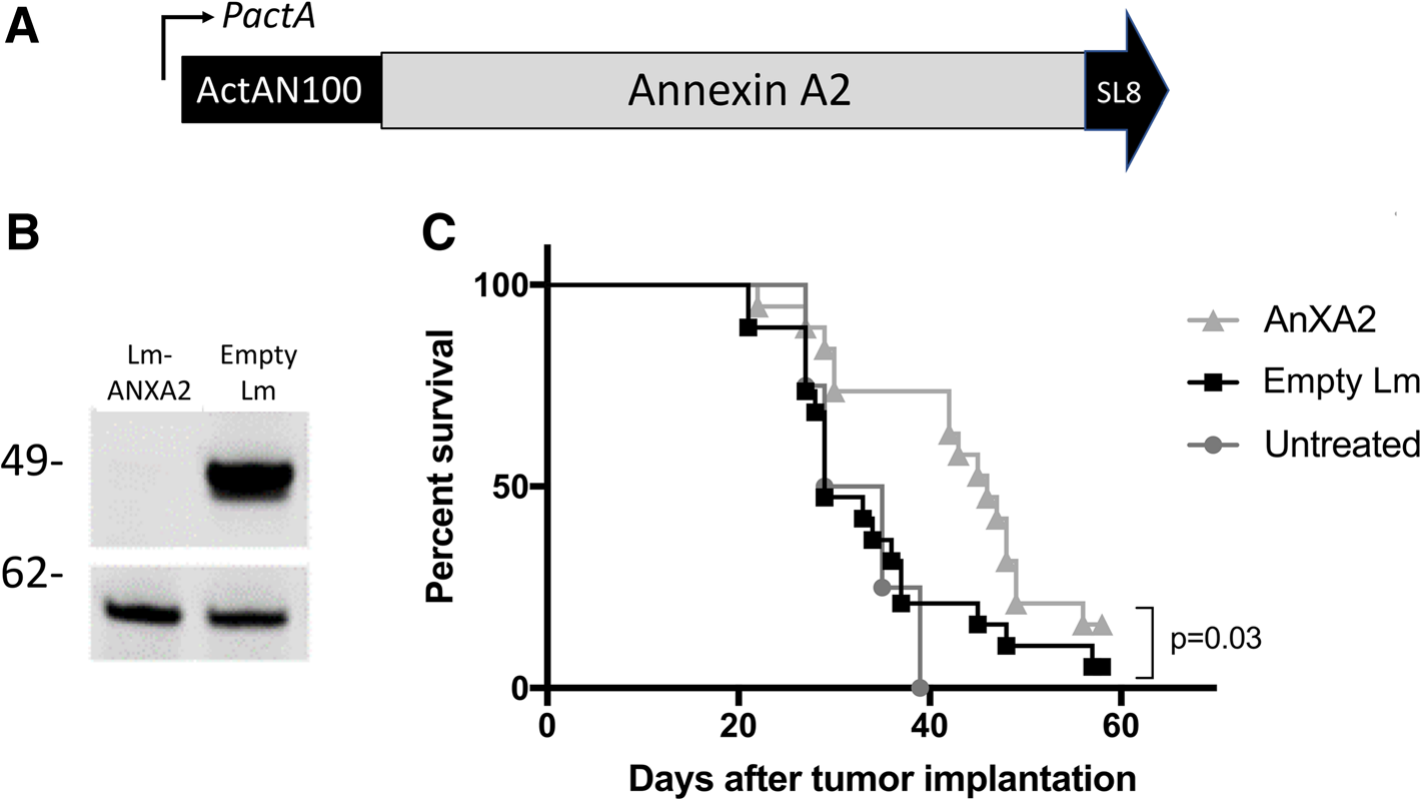
Construction of Lm-ANXA2 and treatment with Lm-ANXA2 improves survival in a PDAC mouse model. **a** Schematic diagram of the LADD strain with the Annexin A2 expression cassette. **b** Western blot analysis of DC2.4 cells infected by Empty Lm and Lm-ANXA2. Top panel: blot with an anti-ActA antibody detecting the ANXA2 fusion protein; bottom panel: blot with anti-Listeria P60 protein as an infection and loading control. Molecular weight showed on the left. **c** Kaplan-Meier survival curves of mice that were implanted with PDAC cells and were untreated (*n* = 5) or treated with either Empty Lm (*n* = 13) or Lm-ANXA2 (*n* = 13). A representative of three repeated experiments is shown

The full-length coding sequence of human ANXA2 (Additional file 1: Figure S1) was cloned into the LADD strain. Expression of ANXA2 is driven by the Listeria promoter ActA that is induced up to 200-fold in the context of the intracellular milieu of the infected antigen presenting cell [26] (Fig. 1a). To test ANXA2 expression in Lm-ANXA2, DC2.4 dendritic cell line was infected with either Lm-ANXA2 or the empty LADD platform strain. Expression of ANXA2 was stable and robust from the LADD platform (Fig. 1b).

### Therapeutic Administration of lm-ANXA2 improves survival in a PDAC mouse model

**Fig. 2:**
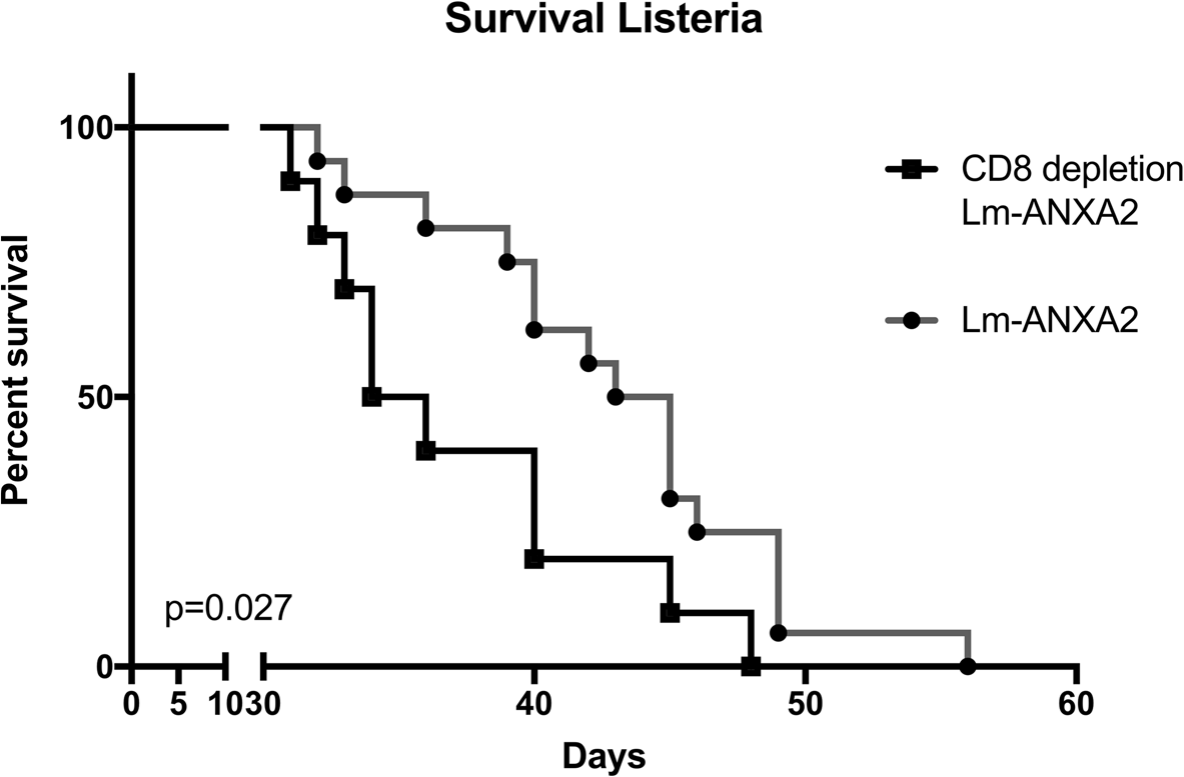
Listeria based Immunotherapy with concomitant CD8+ T cell depletion. Mice implanted with KPC PDAC cells on day 0 underwent treatment with Lm-ANXA2 as described (*n* = 10–12 mice per group). CD8+ depletion was started on day 2 via anti-CD8a antibody (10 mg/kg IP; 2.43, BioXcell) until day 30. Kaplan-Meier curves reveal survival in PDAC bearing mice treated with Lm-ANXA2 and CD8+ T cell depletion is significantly shorter than that of mice treated with Lm-ANXA2 without CD8+ T cell depletion (*p* = 0.027)

We then tested whether targeting ANXA2 with a Listeria-based therapeutic cancer immunotherapy would improve survival in a preclinical model of metastatic PDAC. PDACs developed by the genetically engineered KPC mice (with *KRAS* and *p53* mutations) [24] resemble human PDACs in their pathogenesis and immunobiology and are as difficult as human PDACs to treat by immunotherapeutic agents [32]. First, a previously reported experimental murine model of liver metastases, in which KPC tumor cells are injected directly into the spleen, was employed [30]. The Empty Lm strain was used as a control which can still elicit the innate immune stimulation. Lm-ANXA2 and Empty Lm were administered on days 3, 10, and 17. A single low dose of Cy was given on day 2 for T regulatory cell depletion as reported for other preclinical models of vaccine treatment [8]. Lm-ANXA2 improved the survival of mice compared to the Empty Lm control (*p* = 0.026) (Fig. 1c). Neither Lm-ANXA2 nor Empty Lm caused weight loss, significant transaminitis based on normal serum values in C57Bl/6 mice, or any other evident toxicity in mice, similar to previously described listeria-based immunotherapy [29, 33, 34] (Additional file 1: Figure S2). Previous data suggest the Listeria based immunotherapy response is completely mediated by CD8+ T cells [26]. To reaffirm this in our model, CD8+ T cell depletion was utilized. No statistically significant difference in survival was appreciated in mice with CD8+ T cell depletion treated with Lm-ANXA2 or mice with CD8+ T cell depletion treated with Empty Lm (Additional file 1: Figure S3). Mice with intact CD8+ T cells treated with Empty Lm did not have superior survival compared to CD8+ T cell depleted mice treated with Empty Lm (Additional file 1: Figure S3). Tumor bearing mice treated with Lm-ANXA2 without CD8+ T cell depletion had significantly superior survival than those mice treated with Lm-ANXA2 and concurrent CD8+ T cell depletion throughout the treatment period (Fig. 2), suggesting that the anti-tumor effect of Lm-ANXA2 is mediated by CD8+ T cells.

### Lm-ANXA2 sensitizes PDAC for anti-PD-1 based combinational therapy improving survival outcomes in a mouse model

**Fig. 3:**
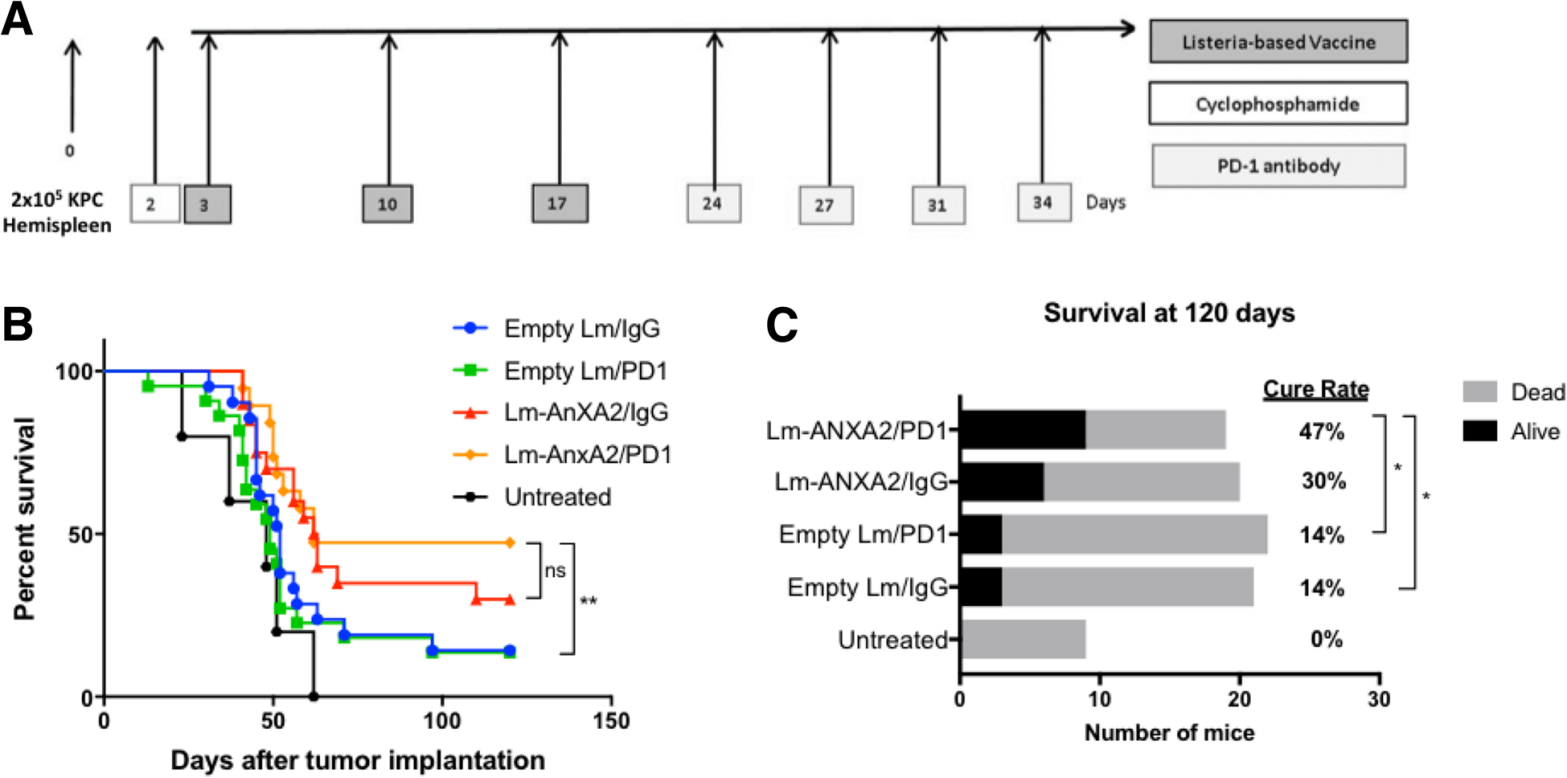
Sequential combination therapy with Cy/Lm-ANXA2 and PD-1 blockade improves survival and cure rate in a PDAC mouse model. **a** Schema of tumor implantation by the hemispleen procedure and treatment with Cy, Empty Lm or Lm-ANXA2, and anti-PD-1 antibody as indicated. **b** Kaplan Meier survival curves and (**c**) cure rate at day 120 of mice implanted with PDAC cells and treated with different combinations of Cy, Empty Lm, Lm-ANXA2, and the anti-PD-1 antibody. Untreated, (*n* = 8); Empty Lm/IgG, (*n* = 21); Empty Lm/anti-PD-1, (*n* = 22); Lm-ANXA2/IgG, (*n* = 19); Lm-ANXA2/anti-PD-1, (*n* = 18). Note that the results combined two independently repeated experiments and therefore have different numbers of mice in each treatment group

The anti-tumor efficacy of Lm-ANXA2 still appears to be modest (Fig. 1c). Thus, we examined whether Lm-ANXA2 can augment the anti-tumor activity of PD-1 inhibitor therapy in the PDAC hemispleen model providing additional benefit if used in combination. Lm-ANXA2 and Empty Lm were administered on days 3, 10, 17 after an initial dose of cyclophosphamide the day prior to the first vaccination (Fig. 3a). Rat anti-mouse PD-1 monoclonal antibody or IgG isotype control were administered biweekly starting on day 24 until death or Day 90. We first found that the concurrent combination of Lm-ANXA2 and anti-PD-1 antibody (data not shown) did not have enhanced anti-tumor activity comparing Lm-ANXA2 alone. Similarly, anti-PD-1 was not effective as single agent therapy in this model. However, we found that sequential combination treatment with Lm-ANXA2 followed by anti-PD-1 antibody improved survival when compared to treatment with Empty Lm alone or Empty Lm + anti-PD-1 blockade antibody (*p* = 0.007) (Fig. 3b). The combination of anti-PD-1 antibody with Empty Lm did not improve survival when compared to treatment with Empty Lm alone (*p* = 0.55) with poor outcomes in both treatment groups. There was a trend toward improved survival with Lm-ANXA2 + anti-PD-1 combination therapy compared to Lm-ANXA2 alone but statistical significance was not reached (*p* = 0.399). It should be noted that Lm-ANXA2 alone, or with IgG isotype control, did not appear to consistently result in long term survival greater than 120 days in this hemispleen mouse model (Fig. 1c; Fig. 3b and c), presumably dependent on the variation of tumor burden established in each individual mouse. Nevertheless, Lm-ANXA2 followed by anti-PD-1 antibody consistently led to long term survival greater than 120 days in approximately 50% of the mice. Alternatively, anti-PD-1 antibody or Empty Lm alone resulted in only an approximately 10% long term survival rate in this mouse model. A modest anti-tumor activity with the Lm-empty/control IgG treatment is consistent with previously published studies showing that the innate immune response induced by Empty Lm has an anti-tumor effect [13, 24]. However, the combination of anti-PD-1 blockade and Empty Lm treatment did not significantly affect the long-term survival rate (*p* = 0.65) (Fig. 3c). The sequential combination treatment of Lm-ANXA2 + anti-PD-1 blockade demonstrated a statistically significant improvement of the long-term mouse survival rate compared to Empty Lm alone or in combination with anti-PD-1 blockade (*p* = 0.02 and p = 0.02, respectively) (Fig. 3c). As similarly seen with the overall survival data, a trend toward an improved cure rate with Lm-ANXA2 + anti-PD-1 combination therapy compared to Lm-ANXA2 alone was appreciated (*p* = 0.22).

### Treatment with lm-ANXA2 and PD-1 blockade in genetically engineered KPC mice with spontaneous PDAC tumors resulted in greater maximal tumor response

**Fig. 4:**
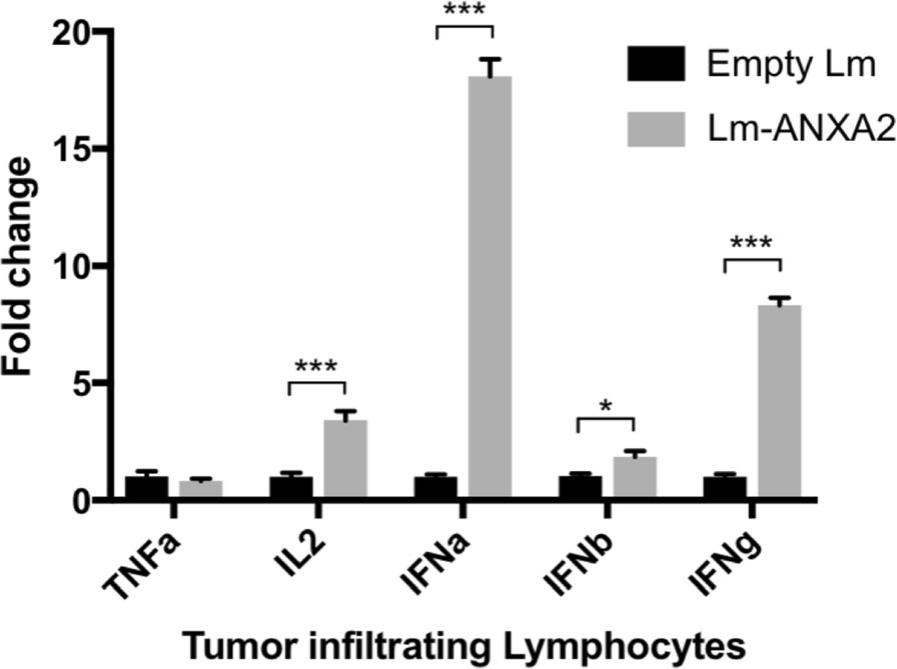
Cytokine expression of infiltrating immune cells in the tumor microenvironment of PDAC following Lm-ANXA2 treatment. Gene expression analysis via qPCR of inflammatory cytokines of tumor infiltrating immune cells isolated from spontaneously developed PDACs in the genetically engineered KPC mice (*n* = 2) following treatment with Empty Lm vs. Lm-ANXA2 . * = *p* < 0.05; *** = *p* < 0.001

**Fig. 5:**
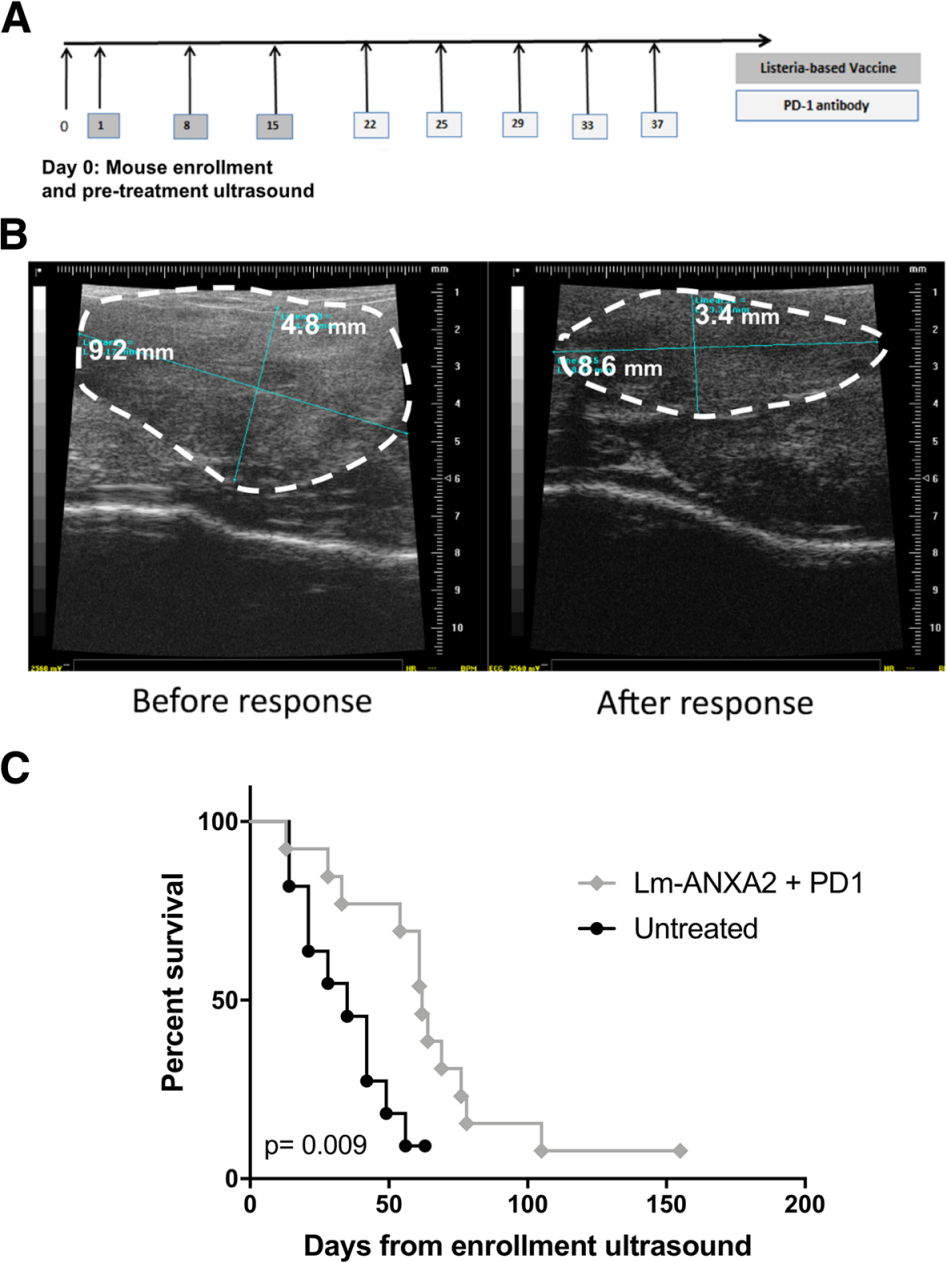
Sequential treatment with Lm-ANXA2 and anti-PD-1 blockade antibody in genetically engineered KPC mice with spontaneous PDAC tumors resulted in prolonged survival. **a** Schema of treatment of genetically engineered KPC mice. **b** Representative ultrasound images of tumors before and after responding to the treatment with Lm-ANXA2/anti-PD-1 antibody. **c** Kaplan Meier survival curves of KPC mice treated with Lm-ANXA2 followed by anti-PD-1 antibody (*n* = 12) and control KPC mice (*n* = 8)

We further examined whether the sequential combination treatment of Lm-ANXA2 and anti-PD-1 blockade therapy can improve survival in PDAC using a genetically engineered murine model of spontaneous PDAC. We first confirmed that RNA expression of multiple anti-tumor cytokines including IL-2, IFNα, IFNβ, and IFNγ, were induced in tumor infiltrating immune cells from PDACs in the transgenic KPC mice treated with Lm-ANXA2 compared to Empty Lm (Fig. 4), further supporting that Lm-ANXA2 can reprogram the TME in KPC mice and potentially make KPC mice more sensitive to the anti-PD-1 antibody treatment. Notably, when compared to untreated mice, Empty Lm did not significantly induce IL-2, IFNα, IFNβ, and IFNγ which were then all induced with Lm-ANXA2 treatment (Additional file 1: Figure S4). The addition of anti-PD-1 therapy to Lm-ANXA2 resulted in a similar increase in expression of the anti-tumor cytokines of interest within infiltrating immune cells as compared to Lm-ANXA2, with a significant increase in IFNβ (Additional file 1: Figure S4). Thus, KPC mice with identified 3-5 mm pancreatic tumors, were treated with Empty Lm vaccination or Lm-ANXA2 followed by anti-PD-1 antibody, respectively (Fig. 5a), and were examined by a small animal ultrasound (Vevo770) weekly (Additional file 1: Figure S5). Weekly tumor volumes were also measured by ultrasound on untreated genetically engineered KPC mice. Representative ultrasound figures are presented (Fig. 5b). By Day 21, when all of the planned listeria treatments had been completed, there was no noticeable difference in tumor response measured by ultrasound between the Lm-ANXA2 and Empty Lm treatment groups. Thus, anti-PD-1 antibody treatment was initiated in the Lm-ANXA2 treatment group on Day 22. Considering the difficulty in breeding large number of the genetically engineered KPC mice and randomizing mice with both equivalent tumor size and age (a critical variable for mouse survival experiments) into multiple treatment groups, we therefore only compared combination treatment to untreated control mice for the survival experiment. As shown in Table 1, 80% of mice in the Lm-ANXA2 treated group had tumor shrinkage after anti-PD-1 antibodies were initiated on Day 22, whereas only 18% of mice in this group had tumor shrinkage before anti-PD-1 antibodies were given (*p* = 0.009). Neither mice in the Empty Lm-treated group nor the untreated group had significant tumor shrinkage before or after Day 22. Maximal tumor response was measured as the volume change from the peak tumor size to the lowest post-peak tumor volume or considered to be “zero” if there was no tumor shrinkage. This value was then compared between treated mice and with control mice (untreated KPC mice). There was a statistically significant increase in maximum tumor response in treatment with the combination therapy of Lm-ANXA2 + anti-PD-1 blockade when compared to no treatment (*p* = 0.022) and to treatment with Empty Lm alone (*p* = 0.027) (Additional file 1: Figure S6). There was no significant difference in maximal tumor response between no treatment and treatment with Empty Lm alone (*p* = 0.974). KPC mice treated with the sequential combination therapy of Lm-ANXA2 + anti-PD-1 blockade had a significantly prolonged survival comparing to the control KPC mice (Fig. 5c). This study for the first time shows that a vaccine-based immunotherapy and PD-1 blockade based combinational immunotherapy is able to result in both objective tumor response and prolonged survival in mice with spontaneously developed PDAC tumors. As ultrasound examination was labor intensive, we limited this experiment to three treatment groups. The observed tumor response unlikely resulted from anti-PD-1 antibody, as KPC mice have been shown to be resistant to single agent anti-PD-1 antibody treatment [35, 36]. Nevertheless, future experiments are warranted to compare between the combination of Lm-ANXA2 and anti-PD-1 antibody and anti-PD-1 antibody alone.

**Table 1:** Sequential treatment with Lm-ANXA2 and anti-PD-1 blockade antibody in genetically engineered KPC mice with spontaneous PDAC tumors resulted in greater tumor response

Treatment Group	Interval decrease in tumor size before day 22	*P* value
Lm-ANXA2	2/11 (18%)	*Reference*
Untreated	1/10 (10%)	NS
Empty Lm	2/12 (17%)	NS
Treatment Group	Interval decrease in tumor size after day 22	*P* value
Lm-ANXA2 + anti-PD-1	8/10 (80%)	*Reference*
Untreated	1/8 (13%)	*p* = 0.015
Empty Lm	2/11 (18%)	*p* = 0.009

Percentages of mice with interval decreases in tumor size measured by ultrasound before and after Day 22 in untreated KPC mice (n = 10), KPC mice treated with Empty

Lm (*n* = 12), and KPC mice treated with Lm-ANXA2 followed by anti-PD-1 antibodies (*n* = 12). ** *p* < 0.01; ***p < 0.001; ns, not significant. Comparison between percentages of mice with interval decreases in tumor size before vs. after Day 22 has a *p* value of 0.009

Immunohistochemistry for ANXA2 was performed on KPC tumors and demonstrated the heterogeneity of ANXA2 expression in PDACs, varying from low to high expression (Additional file 1: Figure S7). This variability in ANXA2 expression may explain why only a portion of mice responded to treatment.

### Combinatorial treatment with lm-ANXA2 and PD-1 blockade increases the antigen-specific T cell response in the tumor microenvironment

We can detect Lm-ANXA2 induced, ANXA2-specific CD8+ T cells in the splenocytes of mice treated with Lm-ANXA2. To identify the CD8+ T cell epitopes, the ANXA2 overlapping peptides were synthesized and initially divided into 4 groups of pooled 15-mer overlapping peptides (Additional file 1: Table S1). In the meantime, we determined whether combination treatment with Lm-ANXA2 and anti-PD-1 blockade increases ANXA2-specific T cell response in the tumor microenvironment.

**Fig. 6:**
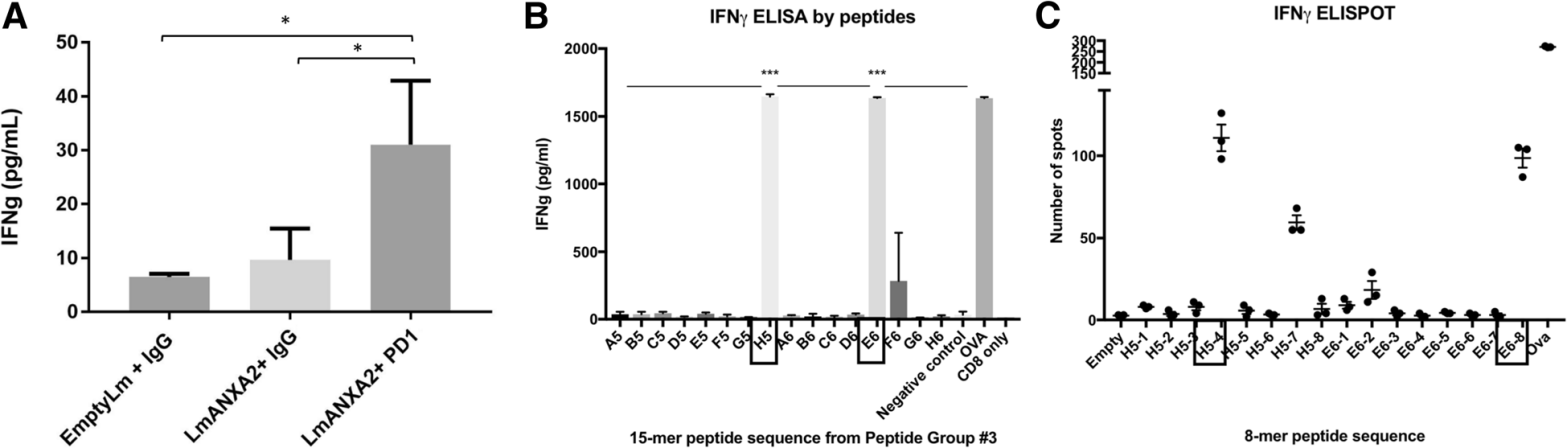
Combinatorial treatment with Lm-ANXA2 and anti-PD-1 blockade antibody increases the ANXA2 epitope-specific T cell response in the tumor microenvironment. **a** CD8+ T cells were isolated and purified from livers on day 28 after hemispleen implantation of KPC tumor cells. Tumor-bearing mice were treated with Cy, either Empty Lm or Lm-ANXA2, and either anti-PD-1 antibody or IgG as in prior study schemes. IFNγ ELISA assays were performed on supernatants collected from CD8+ T cells cocultured with Kb T2 cells exposed to Peptide Group #3. There is a significant difference between combination treatments of Lm-ANXA2 and anti-PD-1 blockade versus Lm-ANXA2 and IgG (*p* = 0.0389). **b** Mice were treated with Lm-ANXA2 on days 1 and 8 and were sacrificed on Day 10. Splenocytes were processed and CD8 cells isolated by negative selection. Kb T2 cells were pulsed with individual 15-mer peptides from ANXA2 Peptide Group #3 as well as positive and negative control peptides. IFNγ ELISA was run on supernatant 18 h following the co-culture of T2 and CD8 T cells. Two epitopes with H5 and E6 peptides were identified with highest expression of IFNγ thus most likely to contain the MHC class I restricted epitopes on ANXA2 protein. **c** IFNγ ELISPOT assays were performed on supernatants collected from CD8+ T cells co-cultured with Kb T2 cells stimulated with individual 8-mer peptide sequences of the active 15-mers H5 and E6, each overlapping by one amino acid. The sequences H5–4: YDAGVKRK and E6–8: VFDRYKSY appear to be the CD8+ T cell ANXA2 epitopes of interest. Each experimental group consisted of 5 mice, pooled, and analyzed individually in triplicates. Data represent mean + SEM from one representative experiment that was repeated once. * *p* < 0.05, *** *p* < 0.001

A priming effect of Lm-ANXA2 treatment was anticipated as supported by the increases in the TME expression of type I interferons (Fig. 4) known to facilitate CD8+ T cell anti-tumor function [37]. Due to the specific role of CD8+ T cell IFNγ production for antitumor activity, corresponding ELISA and ELISPOT were then utilized [38]. CD8+ T cells were isolated and purified from liver on day 28 after hemispleen implantation of KPC tumor cells. Tumor-bearing mice were treated with Cy, either Empty Lm or Lm-ANXA2, in context of either anti-PD-1 antibody or IgG as described in prior study schemes. IFNγ ELISA assays were performed on supernatants collected from CD8+ T cells co-cultured with Kb or Db T2 cells that were exposed to one of four peptide pools of human ANXA2, respectively. Each experimental group consisted of 5 mice and was analyzed individually in triplicates. Peptide pools, instead of individual epitopes, were here focused, considering that T cells for strong epitopes may not necessarily be able to enter the tumors. A rather low IFNγ signal was observed in the Lm-ANXA2 alone treatment group compared to the Empty Lm treatment group (Additional file 1: Figure S8). This suggests that Lm-ANXA2 alone may have a limited capability of inducing antigen-specific, IFNγ expressing T cell infiltration into the tumor microenvironment. However, IFNγ expression of CD8+ T cells exposed to Kb T2 cells stimulated with Peptide Pool #3 was significantly increased with combination treatment of Lm-ANXA2 and anti-PD-1 blockade versus Lm-ANXA2 alone (*p* = 0.0389) (Fig. 6a).

We then further confirmed that there are CD8+ T cell epitopes within Peptide Pool Group #3. Overlapping 15-mer peptides within the Peptide Pool Group #3 were isolated and individually tested to more specifically identify the MHC class I restricted epitopes on ANXA2 with the highest IFNγ activity (Fig. 6b). Two individual 8-mer peptides (YDAGVKRK and VFDRYKSY) from the active 15-mer peptides induced significant IFNγ responses in CD8+ T cells by the ELISPOT assay, suggesting the presence of CD8+ T cell epitopes in the protein region covered by Peptide Pool #3(Fig. 6c).

Other peptide groups did not appear to induce IFNγ expression of CD8+ T cells from mice treated with the combination of Lm-ANXA2 and anti-PD-1 antibody, when exposed to either Kb or Db T2 cells (Additional file 1: Figure S8). Peptide Pool #4 was able to induce IFNγ expression of CD8+ T cells exposed to Kb T2 cells, though, statistical significance was not reached (Additional file 1: Figure S8), suggesting further examination is required. This data suggests that the combination of Lm-ANXA2 and anti-PD-1 blockade treatment induced an ANXA2-specific T cell response in the tumor microenvironment of PDACs and indirectly suggests that the ANXA2-expressing Listeria can induce the production of IFNγ-expressing CD8+ T cell infiltration in PDACs in presence of anti-PD-1 antibody. However, the effect of anti-PD-1 antibody appears to be specifically exerted on T cells for certain Kb-restricted epitopes of ANXA2. We cannot exclude other possibilities including that the repertoire of T cells recognizing Kb-restricted ANXA2 epitopes is larger than that of T cells recognizing Db-restricted epitopes. It remains to be interesting to identify the exact epitopes on ANXA2 that provokes such a response.

## Discussion

This study describes the development of the first human ANXA2-targeted, vaccine-type immunotherapy and its anti-tumor effects in the setting of combination immunotherapy for PDAC. ANXA2 is known to have multiple important roles in the development of many malignancies and was shown by our group to be essential for PDAC metastasis development [19, 21–23, 39, 40]. Thus, ANXA2-expressing, Listeria-based immunotherapy targets a biologically important molecule for cancer development. This is the first time that a cancer vaccine-based immunotherapy has shown to induce an epitope-specific CD8+ T cell response in the tumor microenvironment of a “cold” tumor such as PDAC. The optimal activation of ANXA2 epitope specific T cells in the tumor microenvironment may be reliant on anti-PD-1 antibodies. This is also the first study suggesting that despite its ineffectiveness as a single agent, anti-PD-1 antibody in combination with a vaccine therapy is now able to enhance the IFNγ-expressing, epitope-specific T cells in the tumor microenvironment of a “cold” tumor such as PDAC. Compared to Empty Lm, Lm-ANXA2 as a single agent prolongs survival in the PDAC mouse model; however, the combination of anti-PD-1 antibody and Lm-ANXA2 can increase both survival and the cure rate (> 120 days survival) comparing to Empty Lm or anti-PD-1 antibody alone. Thus, this study further substantiated our previous finding showing that vaccine therapy can prime the PDAC tumor microenvironment for anti-PD-1 antibody treatment. Taken together, this study supports further developing Lm-ANXA2 as a therapeutic agent in combination with anti-PD-1 antibody for pancreatic cancer as well as other cancer types.

The data showing the efficacy of Lm-ANXA2 in sequential combination with anti-PD-1 antibody in the genetically engineered KPC mice with spontaneously developed PDACs are encouraging. Other vaccine-based immunotherapies such as ENO1 and Listeria-KRAS have shown prolonged survival in the aggressive spontaneous KPC mouse model [10, 41]. However, in these studies treatments were administered as early as 4 weeks of age, prior to known invasive tumor development. Therefore, it is uncertain if the effect of these prior vaccines was treatment effect or delayed development of the impending invasive lesion. In our study all KPC mice were 4 months of age or older, with at least 3-5 mm tumors confirmed by ultrasound prior to treatment, thus more directly displaying treatment effect. Previous work from our group also observed that tumor cell vaccines administered before tumor implantation in murine tumor transplant models induced complete protection against the formation of tumors and that this tumor protective effect largely decreased if the vaccines were administrated after tumor implantation [31, 42, 43]. Therefore, the observation in this current study supported a role of Lm-ANXA2 and the combination of Lm-ANXA2 and anti-PD-1 antibody in cancer treatment. Nevertheless, a cancer preventive effect of Lm-ANXA2 cannot be ruled out. Lm-ANXA2 may also potentially serve as a preventative vaccine if utilized in a prophylactic setting.

The treatment plan in this experiment was pre-defined and was limited by the ability to perform the labor-intense ultrasound examination. Therefore, limited doses of anti-PD-1 antibody were given to the mice in this experiment. Although KPC mice treated with Lm-ANXA2 followed by anti-PD-1 antibody demonstrated a statistically significant improvement in the anti-tumor effect comparing to untreated KPC mice, all of the mice, treated or untreated, eventually had disease progression and died. However, it is possible that continuous dosing of anti-PD-1 antibodies may cure some of the KPC mice, which should be tested in future studies. Additionally, heterogeneity of ANXA2 expression may affect the antitumor activity of Lm-ANXA2 and thus would be of interest for future investigation. Nevertheless, this study represents one of only few that demonstrate both objective response and survival benefit to an immunotherapy strategy in this spontaneous pancreatic tumor mouse model that best resembles human PDAC.

In this study, we found that only the sequential combination of Lm-ANXA2 followed by anti-PD-1 antibody, not the concurrent combination of Lm-ANXA2 and anti-PD-1 antibody (data not shown), had enhanced anti-tumor activity compared to Lm-ANXA2 alone. It is possible that the priming effect of Lm-ANXA2 on the tumor microenvironment is important for sensitizing PDACs for anti-PD-1 antibody and that it would require multiple doses of Lm-ANXA2 to prime PDACs for the anti-PD-1 antibody treatment. Previously, we showed that GVAX treatment converts “non-immunogenic” PDACs into “immunogenic” neoplasms by inducing infiltration of T cells in the tumor microenvironment with upregulation of the PD-1–PD-L1 pathway, suggesting that vaccine therapy may sensitize PDACs for immune checkpoint inhibitor therapies [7, 44]. Interestingly, we did not observe the enhancement of anti-tumor activity of Empty Lm with the addition of anti-PD-1 antibody. In addition, there is only a trend of improvement with survival rates from 30 to 47% by adding anti-PD-1 antibody to Lm-ANXA2. By contrast, we observed a significantly larger benefit of adding Lm-ANXA2 to anti-PD-1 antibody as compared to adding Empty Lm to anti-PD-1 antibody. It has been well recognized that anti-PD-1 therapy alone is ineffective in PDAC. Our observation of the significant improvement of survival by adding Lm-ANXA2 to anti-PD-1 antibody comparing to adding Empty Lm to anti-PD-1 antibody suggested that the Lm-ANXA2 vaccine enhances anti-PD-1 antibody responsiveness in the preclinical model. This result also suggests that the epitope-specific T cells induced by Lm-ANXA2 may be critical for improving the effects of anti-PD-1 antibody.

Previously, we observed that a whole cell vaccine, GVAX, can induce anti-ANXA2 antibody response in patients who had longer overall survival following GVAX treatment [14]. Anti-ANXA2 antibody has also been shown to prolong the survival in the mouse model of PDAC [14]. These findings suggest a possible role for therapeutic Anti-ANXA2 antibodies and are thus currently being developed. Nevertheless, previously developed ANXA2-targeting agents including anti-ANXA2 antibodies only have anti-metastasis activities, but do not affect primary PDACs [14]. In this study, we showed that the ANXA2-expressing Listeria-based immunotherapy suppresses both primary PDACs and liver metastases, further supporting the notion that ANXA2 is a target for anti-cancer immunotherapy. Moreover, the combination of a T cell based therapy such as the Listeria-based, ANXA2 targeting immunotherapy and an anti-ANXA2 therapeutic antibody may have synergistic effects and would benefit from future testing.

We were able to detect T cells recognizing ANXA2 peptide pools in the splenocytes (data not shown). However, no significant activation of T cells recognizing ANXA2 peptide pools appeared to have occurred within the PDAC tumors, as shown in this study, no significant difference in IFNγ expression was observed between Lm-ANXA2 treated and Empty Lm treated mice. This result suggests that vaccine-based therapy is unlikely sufficient to induce a potent and durable immune response. The combination of cancer vaccine with checkpoint inhibitors should be the preferred treatment strategy for pancreatic cancer.

It should be noted that the ANXA2 antigen that was engineered in the Listeria is of human origin with an intention to develop this ANXA2-expressing Listeria into a therapeutic agent. Human and mouse ANXA2 proteins are 97.6% identical. Thus, the allogenic effect of the human ANXA2 antigen in a PDAC mouse model is anticipated to be minimal. In fact, the major Db responses measured were to peptide group 2, where there only is a single amino acid difference between the human and mouse protein. Nevertheless, it would be interesting to test whether this human ANXA2 expressing Listeria would be able to induce antigen specific T cell response in previously developed ANXA2-knockout KPC mice [19]. In this study, we detected the ANXA2-specific T cell response within the tumor microenvironment in the liver metastases of a transplant tumor model. It remains to be tested whether such an antigen-specific T cell response would be observed in the primary pancreatic tumors of either an orthotopic transplant or a spontaneous tumor model.

CRS-207, the Listeria-Mesothelin, not in combination with immune checkpoint inhibitors, failed to demonstrate its superiority over chemotherapy in patients with metastatic pancreatic cancer who failed at least two prior therapies (communication from Aduro Biotech). However, the combination of CRS-207 and Nivolumab is being tested in a clinical trial of metastatic PDACs [6]. Similarly, our study suggested that the Listeria based immunotherapy alone has limited anti-tumor activity, but sequential combination with anti-PD-1 antibody can significantly increase T cell function and PDAC cure rate. Therefore, the combination of Lm-ANXA2 and anti-PD-1 antibody should be emphasized in the future development of Lm-ANXA2.

## Conclusions

For the first time, a Listeria vaccine-based immunotherapy was shown to be able to induce a tumor antigen-specific T cell response within the tumor microenvironment of a “cold” tumor such as PDAC and sensitize the tumor to checkpoint inhibitor therapy. Moreover, this combination immunotherapy led to objective tumor responses and survival benefit in the mice with spontaneously developed PDAC tumors. Therefore, our study supports developing Lm-ANXA2 as a therapeutic agent in combination with anti-PD-1 antibody for PDAC treatment.

## Additional file

Additional file 1: **Figure S1.** Murine and human ANXA2 protein sequences. Brackets note differences. **Figure S2.** Sequential weights in mice following (A) Empty-Lm or (B) Lm-ANXA2. (C) Serum AST and ALT levels in mice following Empty-Lm or Lm-ANXA2 were measured at SRI Biosciences’ Clinical Analysis Laboratory. 95% CI of normal values notated. ALT was within normal limits despite significant difference in the Lm-ANXA2 group. **Figure S3.** Hemispleen mice underwent treatment with Empty-Lm or Lm-ANXA2 with or without CD8+ depletion. (A) No significant difference in survival comparing untreated mice to mice treated Empty-Lm and CD8+ depletion or mice treated with Lm-ANXA2 and CD8+ depletion. (B) No significant difference in survival in mice treated with Empty-Lm without CD8+ depletion compared to Empty-Lm and CD8+ depletion. **Figure S4.** Gene expression analysis via qPCR of inflammatory cytokines of TIL isolated from spontaneously developed PDACs in KPC mice following treatment with Empty-Lm, Lm-ANXA2 and Lm-ANXA2 + anti-PD-1. No significance comparing Untreated and Empty-Lm in all genes. No significance comparing Lm-ANXA2 and Lm-ANXA2 + anti-PD-1 in all genes, except for IFNβ.** Figure S5.** Ultrasound measurements of (A) untreated, (B) Empty-Lm and (C) Lm-ANXA2+anti-PD-1 treated KPC mice. Day 22 notated. **Figure S6.** Maximal tumor volume reduction from peak tumor size to lowest post-peak tumor size was measured and compared between untreated, Empty-Lm, and Lm-ANXA2+anti-PD-1 treated KPC mice. **Figure S7.** Heterogenic immunohistochemistry expression of ANXA2 in KPC mice: 40% of KPC tumors with low and 60% high expression. **Figure S8.** CD8+ cells were isolated from TIL after hemispleen procedure and co-cultured with peptide-pulsed T2-cells. IFNγ expression of Empty-Lm vs. Lm-ANXA2 exposed to (A) Kb-T2 and (B) Db-T2-cells stimulated with corresponding pooled-peptide groups. ELISA repeated with anti-PD-1 treatment. IFNγ expression of treatment groups exposed to Kb-T2 and (C) peptide group #1, (D) #2 or (E) #4. Db-T2-cells stimulated with (F) peptide groups #1, (G) #2, (H) #3, or (I) #4. **Table S1.** Overlapping peptide groups of ANXA2. (DOCX 4947 kb)

## Abbreviations

ALT: alanine aminotransferase
ANXA2: Annexin A2
AST: aspartate aminotransferase
CRS-207: mesothelin-expressing Listeria-based immunotherapy
Cy: cyclophosphamide
GM-CSF: granulocyte-macrophage colony-stimulating factor
GVAX: GM-CSF-secreting pancreatic tumor whole cell vaccine
IACUC: Institutional Animal Care and Use Committee
IFN: interferon
IP: intraperitoneal
KPC: KrasLSL.G12D/+; p53R172H/+; PdxCretg/+
LADD: Δ*actA* Δ*inlB* live attenuated, double deleted *Listeria* platform strain
Lm-ANXA2: ANXA2-expressing Listeria-based immunotherapy
NCBI: National Center for Biotechnology Information
PBS: phosphate-buffered saline
PD-1: programmed death-1
PDAC: pancreatic ductal adenocarcinoma
PD-L1: programmed death-ligand 1
qPCR: quantitative real-time RT-PCR
TNF: tumor necrosis factor
