# Supplementary material for: Anti-pancreatic tumor efficacy of a Listeria-based, Annexin A2-targeting immunotherapy in combination with anti-PD-1 antibodies
Source: J Immunother Cancer. 2019 May 22;7:132. doi: 10.1186/s40425-019-0601-5 (PMC6529991; doi:10.1186/s40425-019-0601-5)

**Anti-pancreatic tumor efficacy of a Listeria-based, Annexin A2-targeting immunotherapy in combination with anti-PD-1 antibodies**

**Authors:** Victoria Kim^1,2,3^†, Alex Blair^1,2,3,4^†, Peter Lauer^5^, Kelly Foley^1,2^, Xu Che^1,2,4^, Kevin Soares^1,2,3^, Tao Xia^1,2,4^, Stephen Muth^1,2,4^, Jennifer Kleponis^1,2^, Todd Armstrong^1,2^, Christopher Wolfgang^1,2,3,4^, Elizabeth M. Jaffee^1,2,4^, Dirk Brockstedt^5^, Lei Zheng^1,2,3,4*^

†VK and ABB contributed equally to this manuscript

**Affiliations:**

^1^The Sidney Kimmel Comprehensive Cancer Center, The Johns Hopkins University School of Medicine, Baltimore, Maryland, 21287, USA.

^2^Department of Oncology, The Johns Hopkins University School of Medicine, Baltimore, Maryland, 21287, USA.

^3^Department of Surgery, The Johns Hopkins University School of Medicine, Baltimore, Maryland, 21287, USA.

^4^The Pancreatic Cancer Precision Medicine Program of Excellence, The Johns Hopkins University School of Medicine, Baltimore, Maryland, 21287, USA

^5^Aduro Biotech, Inc., Berkeley, California, USA.

***Corresponding Author:**

Lei Zheng, M.D., Ph.D., 1650 Orleans Street, CRB1 Room 488, Baltimore, MD 21042. Tel: 410-5026241, Fax: 410-614-8216, Email: [lzheng6@jhmi.edu](mailto:lzheng6@jhmi.edu)

**Running title:** Annexin A2 expressing Listeria-based immunotherapy

**Supplementary Tables and Figures**

**Figure S1**

hANXA2 MSTVHEILCKLSLEGDHSTPPSAYGSVKAYTNFDAERDALNIETAIKTKGVDEVTIVNIL 60

MSTVHEILCKLSLEGDHSTPPSAYGSVK YTNFDAERDALNIETA+KTKGVDEVTIVNIL

mANXA2 MSTVHEILCKLSLEGDHSTPPSAYGSVKPYTNFDAERDALNIETAVKTKGVDEVTIVNIL 60

hANXA2 TNRSNAQRQDIAFAYQRRTKKELASALKSALSGHLETVILGLLKTPAQYDASELKASMKG 120

TNRSN QRQDIAFAYQRRTKKEL SALKSALSGHLETVILGLLKTPAQYDASELKASMKG

mANXA2 TNRSNVQRQDIAFAYQRRTKKELPSALKSALSGHLETVILGLLKTPAQYDASELKASMKG 120

hANXA2 LGTDEDSLIEIICSRTNQELQEINRVYKEMYKTDLEKDIISDTSGDFRKLMVALAKGRRA 180

LGTDEDSLIEIICSRTNQELQEINRVYKEMYKTDLEKDIISDTSGDFRKLMVALAKGRRA

mANXA2 LGTDEDSLIEIICSRTNQELQEINRVYKEMYKTDLEKDIISDTSGDFRKLMVALAKGRRA 180

hANXA2 EDGSVIDYELIDQDARDLYDAGVKRKGTDVPKWISIMTERSVPHLQKVFDRYKSYSPYDM 240

EDGSVIDYELIDQDAR+LYDAGVKRKGTDVPKWISIMTERSV HLQKVF+RYKSYSPYDM

mANXA2 EDGSVIDYELIDQDARELYDAGVKRKGTDVPKWISIMTERSVCHLQKVFERYKSYSPYDM 240

hANXA2 LESIRKEVKGDLENAFLNLVQCIQNKPLYFADRLYDSMKGKGTRDKVLIRIMVSRSEVDM 300

LESI+KEVKGDLENAFLNLVQCIQNKPLYFADRLYDSMKGKGTRDKVLIRIMVSRSEVDM

mANXA2 LESIKKEVKGDLENAFLNLVQCIQNKPLYFADRLYDSMKGKGTRDKVLIRIMVSRSEVDM 300

hANXA2 LKIRSEFKRKYGKSLYYYIQQDTKGDYQKALLYLCGGDD 339

LKIRSEFKRKYGKSLYYYIQQDTKGDYQKALLYLCGGDD

mANXA2 LKIRSEFKRKYGKSLYYYIQQDTKGDYQKALLYLCGGDD 339​

**Figure S1 Comparison between murine and human ANXA2 protein sequences.** Edited peptide sequences to demonstrate difference between murine and human ANXA2 with brackets.  They are 98% homologous.

**Figure S2**


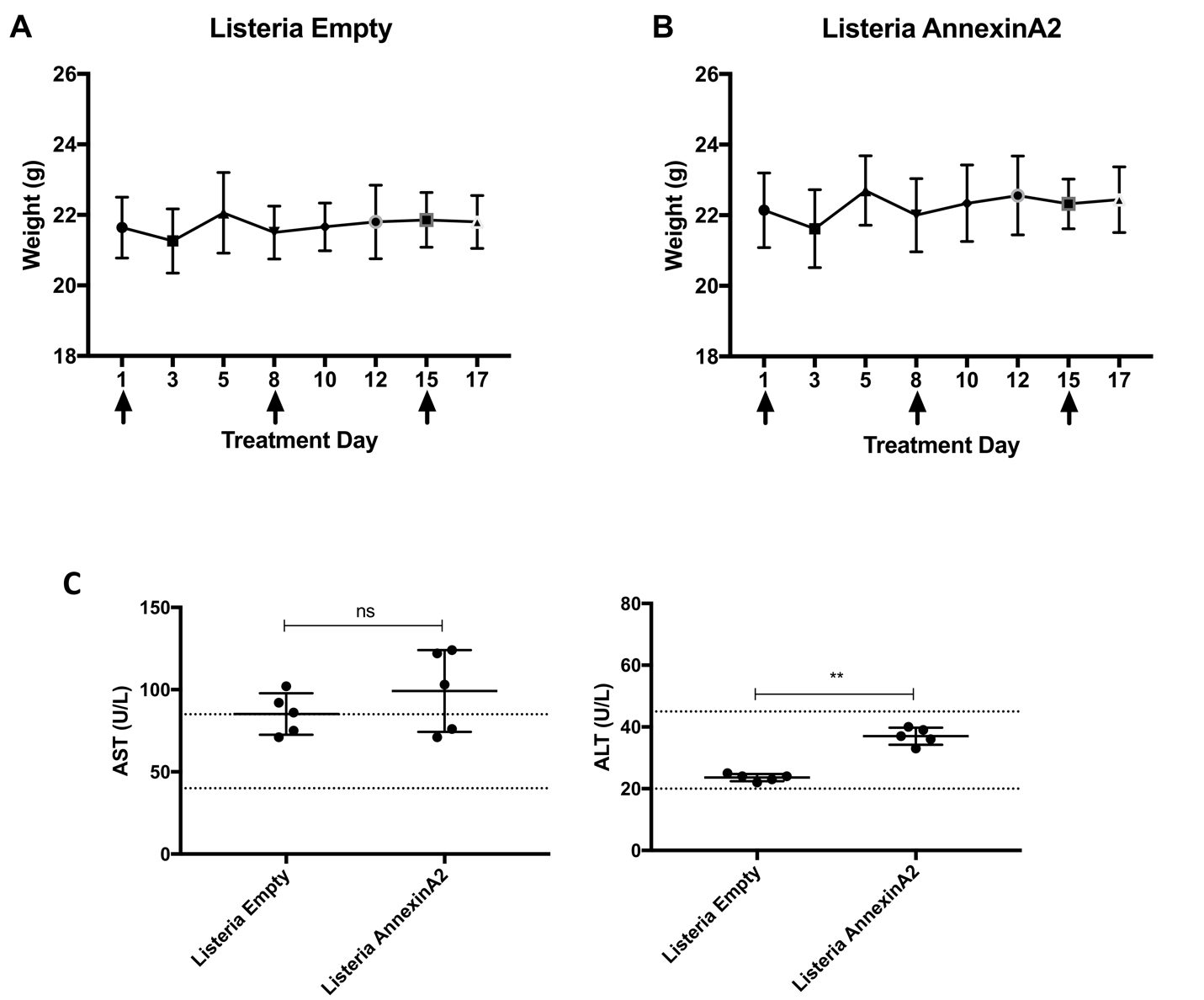


**Figure S2 Toxicity analysis of Lm-ANXA2 and Empty Lm in mice.** Sequential weights of C57Bl/6 mice (n=5) following treatment with Empty Lm (A) or Lm-ANXA2 (B). Weight in grams. Arrows indicate treatment days. (C) Serum AST and ALT levels in C57Bl/6 mice (n=5) following treatment with Empty Lm (Listeria Empty) or Lm-ANXA2 (Listeria AnnexinA2) were measured at SRI Biosciences’ Clinical Analysis Laboratory. Dashed line represents 95% CI of normal serum values in C57Bl/6 mice. ns=not significant; **=p<0.01. Note that ALT in all mice was within normal limit even though it was significantly higher in the Lm-ANXA2 group than the Empty Lm group.

**Figure S3**

**
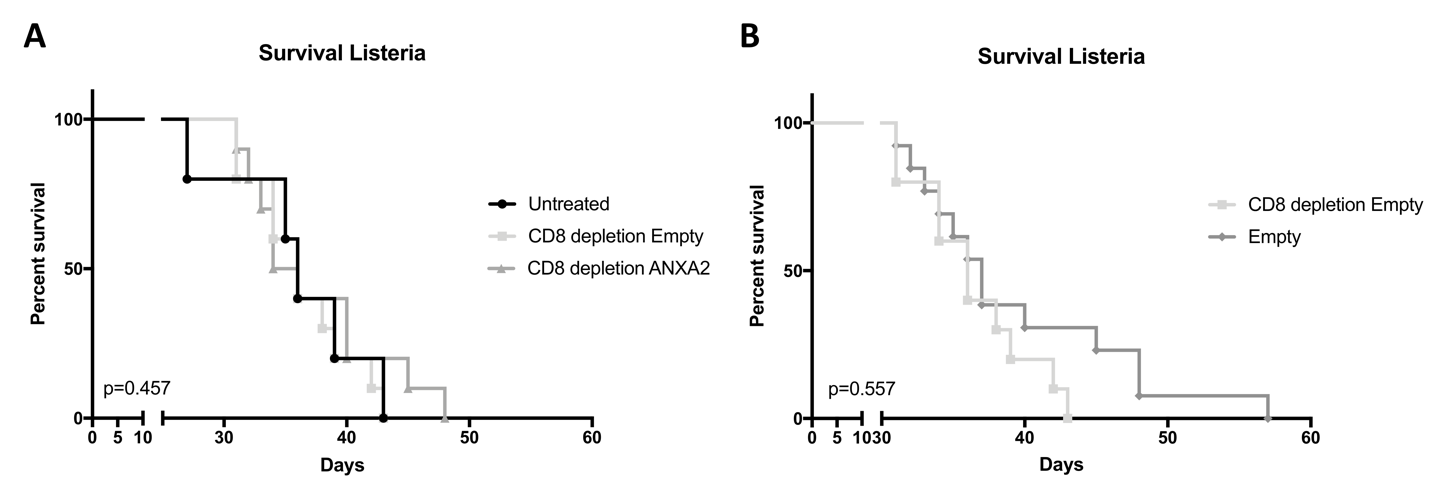
**

**Figure S3. Listeria based immunotherapy with concomitant CD8+ T cell depletion.** Mice implanted with KPC PDAC cells on day 0 underwent treatment with Empty Lm or Lm-ANXA2 as previously described (n=10-12 mice per group). CD8+ depletion was started on day 2 via anti-CD8a antibody (10mg/kg IP; 2.43, BioXcell) until day 30 and was given in both control groups. (A) Kaplan-Meier curves reveal there is no statistically significant difference in survival comparing untreated mice to mice treated with Empty Lm and CD8+ depletion or mice treated with Lm-ANXA2 and CD8 depletion (p=0.457). (B) No statistically significant difference in survival was appreciated in PDAC bearing mice treated with Empty Lm without CD8+ depletion compared to mice treated with Empty Lm and CD8+ T cell depletion (p=0.557).

**Figure S4**

**
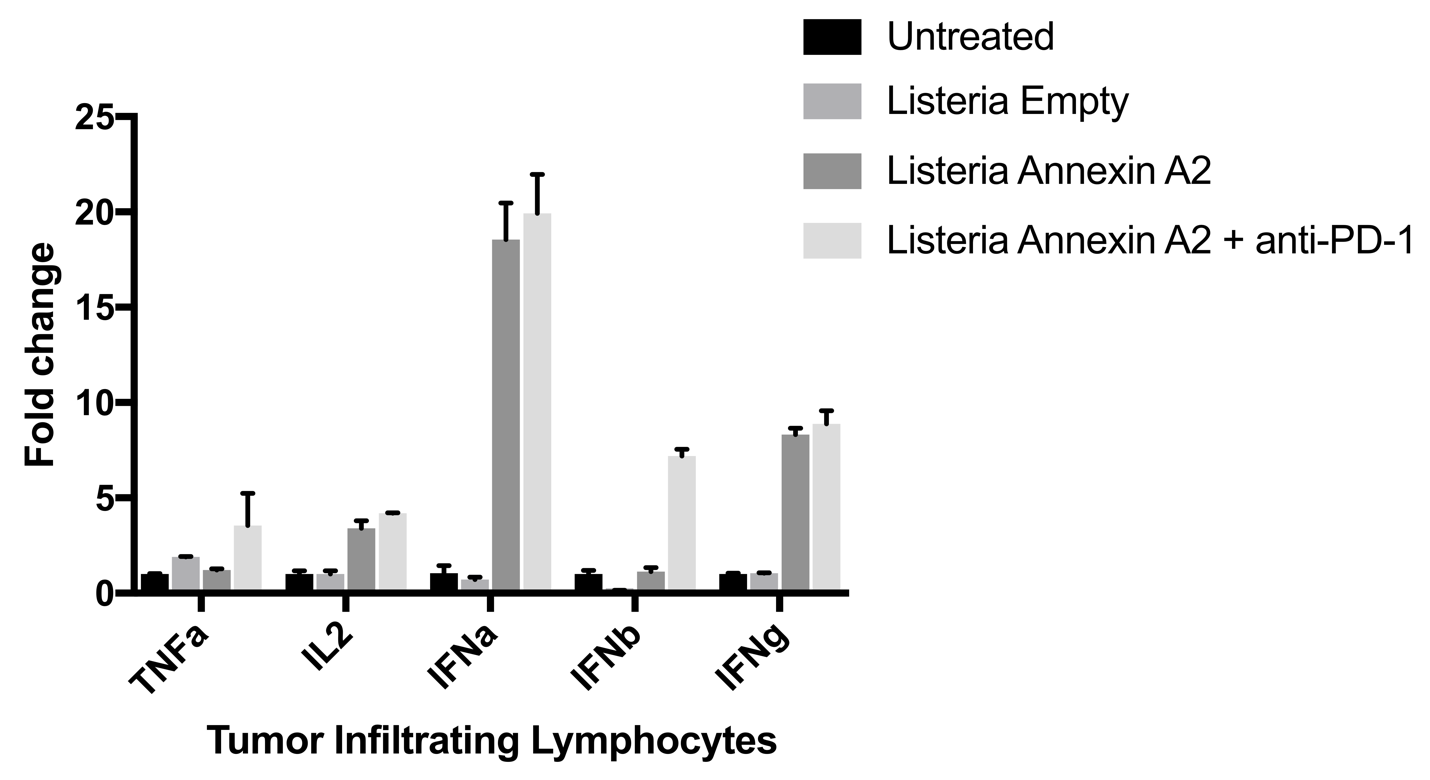
**

**Figure S4. Cytokine expression of infiltrating immune cells in the tumor microenvironment of PDAC following Lm-ANXA2 treatment.** Gene expression analysis via aPCR of inflammatory cytokines of tumor infiltrating immune cells isolated from spontaneously developed PDACs in the genetically engineered KPC mice (n=4) following treatment with Untreated, Empty Lm, Lm-ANXA2 and Lm-ANXA2 + anti-PD-1. No statistical significance is noted comparing Untreated and Empty Lm in all genes of interest. Similarly, no statistical significance is noted comparing Lm-ANXA2 and Lm-ANXA2 + anti-PD-1 in all genes, except for IFNb (p=0.004).

**Figure S5**

**Figure S5. Ultrasound measurement of the PDAC tumors in KPC mice.** (A) Ultrasound measurements of untreated mice, (B) mice treated with Empty Lm and (C) mice treated with Lm-ANXA2 followed by anti-PD1 antibodies. Trend lines indicate Day 22.

**Figure S6**

**
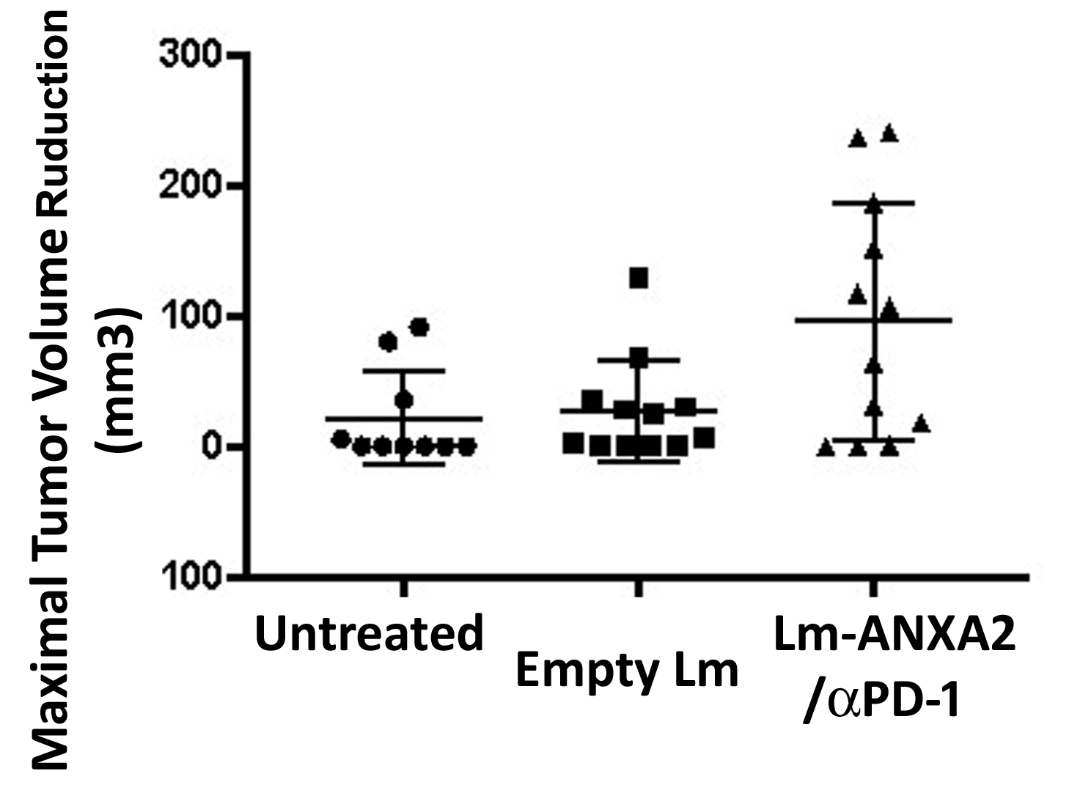
**

**Figure S6. Maximal tumor volume reduction in KPC mice treated with Lm-ANXA2 followed by anti-PD-1 antibodies.** The maximal tumor volume reduction from the peak tumor size to the lowest post-peak size of the tumor was measured and compared between untreated KPC mice (n=10), KPC mice treated with Empty Lm followed by IgG (n=12), and KPC mice treated with Lm-ANXA2 followed by anti-PD-1 antibodies (n=12).

**Figure S7**

**Figure S7. Expression of ANXA2 in KPC tumor.** Immunohistochemistry for ANXA2 was performed in 10 KPC mice. Analysis reveals heterogenic expression of ANXA2, with 40% of KPC tumors having low expression and 60% having high expression.

**Figure S8**

**Figure S8. Examination of T cell response to ANXA2 overlapping peptide groups in the tumor microenvironment.** CD8+ T cells were isolated and purified from livers on day 14 after hemispleen implantation of KPC tumor cells. Tumor-bearing mice were treated with Cy, either Empty Lm or Lm-ANXA2, and either anti-PD-1 antibody or IgG as in prior study schemes. ELISA assays were performed by co-culturing the CD8+ T cells with Kb or Db T2 cells that were exposed to one of four different peptide sequence groups. Each experimental group consisted of 5 mice, pooled, and analyzed individually in triplicates. Data represent mean + SEM from one representative experiment that was repeated twice. IFNγ expression of the treatment groups (Empty Lm vs. Lm-ANXA2) exposed to (A) Kb T2 and (B) Db T2 cells that were stimulated with Peptide Groups #1, #2, #3, or #4, was shown. Additional experiment performed with the addition of either anti-PD-1 antibody or IgG as in prior study schemes were performed with the same ELISA protocol. IFNγ expression of the treatment groups exposed to Kb T2 and (C) peptide group #1, (D) #2 or (E) #4 is shown. Simiarly, Db T2 cells that were stimulated with (F) peptide groups #1, (G) #2, (H) #3, or (I) #4 is shown.

**Supplementary Table S1 Overlapping peptide groups of ANXA2**


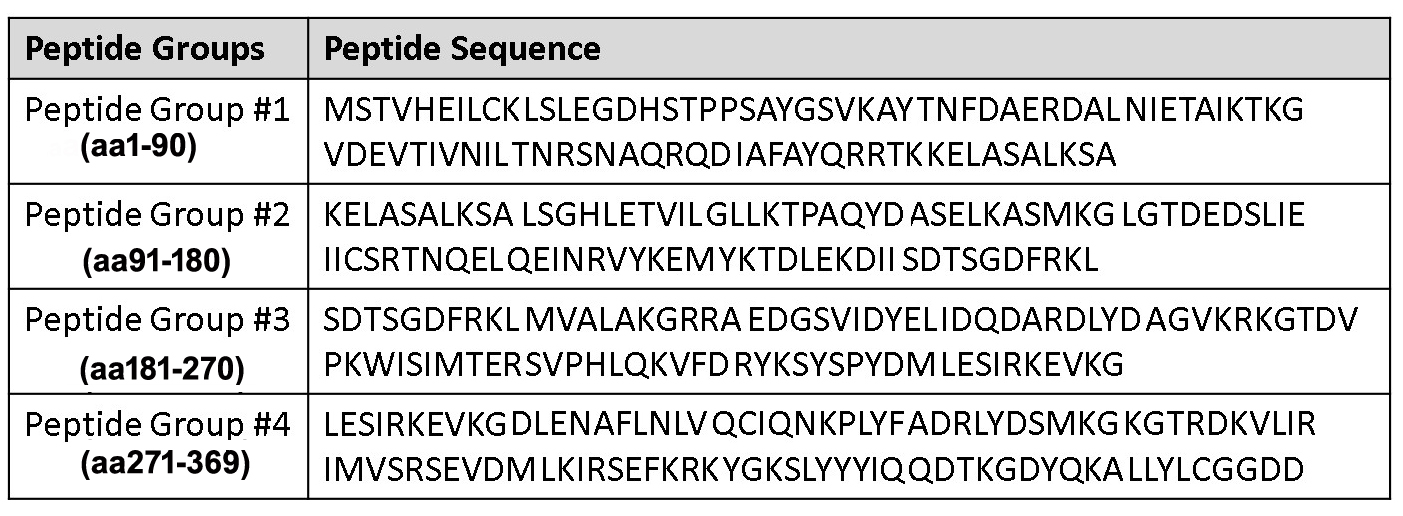

Supplement: Supplementary file 1 — Figure S1. Murine and human ANXA2 protein sequences. Brackets note differences. Figure S2. Sequential weights in mice following (A) Empty-Lm or (B) Lm-ANXA2. (C) Serum AST and ALT levels in mice following Empty-Lm or Lm-ANXA2 were measured at SRI Biosciences’ Clinical Analysis Laboratory. 95% CI of normal values notated. ALT was within normal limits despite significant difference in the Lm-ANXA2 group. Figure S3. Hemispleen mice underwent treatment with Empty-Lm or Lm-ANXA2 with or without CD8+ depletion. (A) No significant difference in survival comparing untreated mice to mice treated Empty-Lm and CD8+ depletion or mice treated with Lm-ANXA2 and CD8+ depletion. (B) No significant difference in survival in mice treated with Empty-Lm without CD8+ depletion compared to Empty-Lm and CD8+ depletion. Figure S4. Gene expression analysis via qPCR of inflammatory cytokines of TIL isolated from spontaneously developed PDACs in KPC mice following treatment with Empty-Lm, Lm-ANXA2 and Lm-ANXA2 + anti-PD-1. No significance comparing Untreated and Empty-Lm in all genes. No significance comparing Lm-ANXA2 and Lm-ANXA2 + anti-PD-1 in all genes, except for IFNβ. Figure S5. Ultrasound measurements of (A) untreated, (B) Empty-Lm and (C) Lm-ANXA2+anti-PD-1 treated KPC mice. Day 22 notated. Figure S6. Maximal tumor volume reduction from peak tumor size to lowest post-peak tumor size was measured and compared between untreated, Empty-Lm, and Lm-ANXA2+anti-PD-1 treated KPC mice. Figure S7. Heterogenic immunohistochemistry expression of ANXA2 in KPC mice: 40% of KPC tumors with low and 60% high expression. Figure S8. CD8+ cells were isolated from TIL after hemispleen procedure and co-cultured with peptide-pulsed T2-cells. IFNγ expression of Empty-Lm vs. Lm-ANXA2 exposed to (A) Kb-T2 and (B) Db-T2-cells stimulated with corresponding pooled-peptide groups. ELISA repeated with anti-PD-1 treatment. IFNγ expression of treatment groups exposed to Kb-T2 and (C) peptide group #1, (D) #2 or (E) # [file 40425_2019_601_MOESM1_ESM.docx]
